# The Fortification of Fruit Mousses with Encapsulated Bioactive Compounds in a Potato Starch Matrix as an Example of Novel Functional Foods

**DOI:** 10.3390/ijms27021106

**Published:** 2026-01-22

**Authors:** Magdalena Krystyjan, Gohar Khachatryan, Karen Khachatryan, Anna Lenart-Boroń, Robert Socha, Zuzanna Potoniec

**Affiliations:** 1 Department of Carbohydrates Technology and Cereal Processing, Faculty of Food Technology, University of Agriculture in Krakow, Al. Mickiewicza 21, 31-120 Krakow, Poland; 2 Department of Food Analysis and Quality Assessment, Faculty of Food Technology, University of Agriculture in Krakow, Al. Mickiewicza 21, 31-120 Krakow, Poland; gohar.khachatryan@urk.edu.pl (G.K.); robert.socha@urk.edu.pl (R.S.); 3 Laboratory of Nanotechnology and Nanomaterials, Faculty of Food Technology, University of Agriculture in Krakow, Al. Mickiewicza 21, 31-120 Krakow, Poland; karen.khachatryan@urk.edu.pl; 4 Department of Microbiology and Biomonitoring, Faculty of Agriculture and Economics, University of Agriculture in Krakow, 30-059 Krakow, Poland; anna.lenart-boron@urk.edu.pl; 5 Food Technologists Scientific Circle, Carbohydrate Technology Section, University of Agriculture in Krakow, Al. Mickiewicza 21, 31-120 Krakow, Poland; zuzanna.potoniec@student.urk.edu.pl

**Keywords:** functional food, encapsulation, potato starch, bioactive compounds, novel foods

## Abstract

The increasing consumption of highly processed foods has resulted in a reduced intake of essential vitamins, minerals, and bioactive compounds, thereby intensifying interest in the development of functional food. This study aimed to enrich fruit mousses with bioactive compounds derived from elderberry extract using an encapsulation strategy. Three formulations were prepared: a control mousse, a mousse enriched via direct addition of the extract, and a mousse supplemented with a nanoemulsion. Comprehensive analyses, including SEM (Scanning Electron Microscopy), FTIR (Fourier Transform Infrared Spectroscopy), colorimetry, texture and rheological measurements, phenolic acid and flavonoid content, antioxidant and reducing activity, sensory evaluation, and microbiological assessment, confirmed the successful formation of submicron capsules (400–900 nm), effective incorporation of grape seed oil into the fruit mousse formulations, and minimal color alteration (ΔE* < 1). The enriched mousses exhibited slightly higher hardness (7.5%) and adhesiveness (5.4%) as well as enhanced antioxidant and reducing activity compared to the control. Rheological analyses indicated improved structural stability resulting from fortification. Sensory evaluation demonstrated good consumer acceptance, while microbiological analyses suggested a potential shelf-life extension due to inhibited microbial growth. Overall, encapsulation proved to be an effective approach for incorporating elderberry-derived bioactive substances into fruit mousses while preserving product quality.

## 1. Introduction

Current developments in the food industry emphasize creating products with enhanced nutritional quality and health-promoting benefits. Increasing attention is being given to functional foods, which provide health benefits beyond basic nutrition [1,2,3]. In response to modern lifestyles and growing consumer expectations, such products are gaining increasing popularity [4]. A well-balanced diet rich in vitamins, minerals, and bioactive compounds supports immune function, particularly during periods of increased susceptibility to infections. Bioactive compounds may enhance the immune system by stimulating antibody production and protecting the body against bacterial and viral infections. Among commonly used natural sources with immunomodulatory properties, black elderberry (*Sambucus nigra*) is of particular interest due to its high content of vitamin C, anthocyanins, and tannins [5,6,7,8,9]. Previous studies have shown that elderberry extract can inhibit influenza A (H1N1) virus replication in vitro, showing dose-dependent antiviral activity [10].

Fruits, vegetables, and their derived products are essential components of a healthy daily diet and contribute to the prevention of numerous chronic diseases, including cardiovascular diseases, osteoporosis, cognitive decline, and certain cancer [11,12,13]. These foods provide dietary fiber, antioxidants (e.g., vitamin C, β-carotene, and flavonoids), and essential minerals that support gastrointestinal function, reduce oxidative stress, and promote overall health [14,15]. In recent years, fruit and vegetable mousses gained popularity as convenient snack products, created mainly to make children’s meals more attractive, which is particularly important because it can encourage young consumers to eat more vegetables and less commonly consumed fruits. Moreover, the fortification of bioactive compounds offers an effective strategy for enhancing immune-supporting properties. Unfortunately, conventional processing methods often result in the degradation of vitamins and phenolic compounds [4,16]. Consequently, traditional fortification approaches may be insufficient, highlighting the need for protective technologies such as encapsulation that protect sensitive bioactive ingredients and improve the nutritional value of the final product [4,17].

Grape seed oil, as a rich source of linoleic acid, also serves as an effective lipophilic carrier of bioactive compounds, including tocopherols, phenolic compounds, and phytosterols, thereby exhibiting antioxidant activity. Due to its neutral taste and odor, grape seed oil is well accepted by consumers. Furthermore, the presence of tocopherols contributes to its relatively high oxidative stability, which is significantly greater than that of flaxseed oil [18].

Encapsulation is used to protect sensitive components from environmental degradation, extend shelf life, and enable controlled release in food products. It also improves sensory quality by masking undesirable tastes and odors and limiting unwanted interactions between ingredients [19,20]. Capsules are generally classified as microcapsules (1–1000 μm) and nanocapsules (<1 μm), with structures ranging from core–shell systems to irregular multi-droplet matrices [21,22,23,24,25,26,27,28]. The selection of an appropriate encapsulating polymer is crucial, as it directly affects the stability, protection, and bioavailability of the encapsulated compounds. In food applications, naturally derived polymers are preferred due to safety, regulatory compliance, and sensory acceptability reasons [29,30].

Numerous studies have shown that starch-based encapsulation effectively protects bioactive compounds, improves their stability, and enables controlled release, particularly in dry or semi-dry food systems [22,31]. However, despite extensive research on starch-based encapsulation, its application in complex, high-moisture food systems such as fruit mousses remains insufficiently explored. Fruit mousse matrices are characterized by high water content, low pH, and the presence of natural sugars, organic acids, and phenolic compounds, which may adversely affect capsule integrity, stability, and release behavior. As a result, the functionality of encapsulated bioactive compounds in such systems is still not fully understood. Compared to widely studied corn or modified starches, potato starch has received relatively limited attention as an encapsulation material. Its high swelling capacity, strong water-binding properties, and high-viscosity gels may enhance capsule stability in high-moisture food products. Moreover, its neutral sensory characteristics, non-allergenic nature, and natural occurrence in fruit-based products, together with its GRAS status, make potato starch an attractive matrix for functional food applications [25,31].

Therefore, the aim of this study is to develop a novel functional food in the form of a health-oriented snack suitable for a broad consumer group, enriched with bioactive compounds derived from elderberry extract and grape seed oil incorporated as micro- and nano-sized capsules. It is hypothesized that potato starch, a naturally occurring component of fruit-based products, can serve as an effective encapsulation matrix, conferring protection and stability to bioactive compounds in fruit mousse systems while maintaining their functional properties and enabling the formulation of a fully natural product.

## 2. Results and Discussion

### 2.1. Physicochemical Properties of Fruit Mousses

#### 2.1.1. Morphology of Fortified Mousse

**Figure 1:**
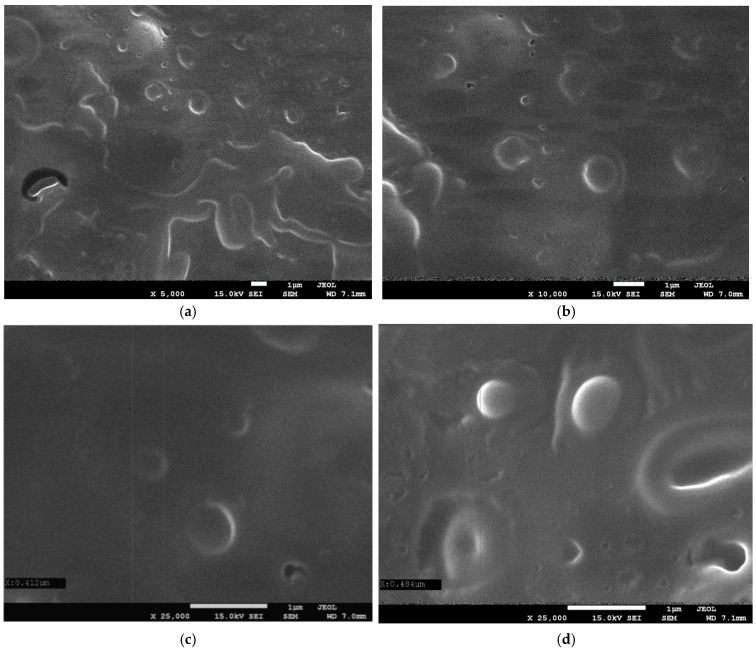
Scanning Electron Microscopy (SEM) micrographs of the dried encapsulated mousse (F2) at different magnifications: (**a**) 5000×, (**b**) 10,000×, (**c**) 25,000×, and (**d**) 25,000×.

Scanning Electron Microscopy (SEM) was used to visualize the morphology of the obtained mousse (F2) containing nanocapsules with bioactive compounds. For the analysis, the sample was first placed in a Petri dish and dried in an oven. The acquired images (Figure 1) revealed the presence of spherical structures dispersed throughout the matrix. These structures, with a size of approximately 400–900 nm, indicate the successful formation of nanocapsules (submicron capsules). The micrographs confirmed that the core material (elderberry extract and oil) was encapsulated by the biopolymer chains, i.e., starch, forming stable capsules.

#### 2.1.2. Structure of Products

**Figure 2:**
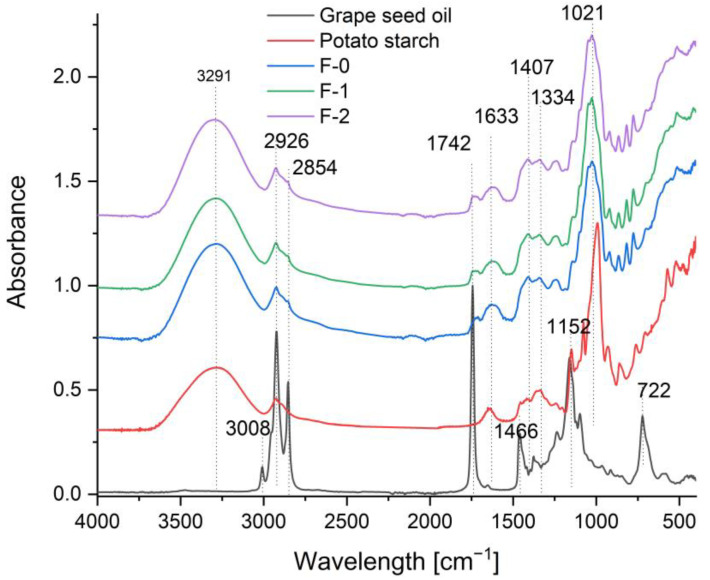
FTIR spectra of the analyzed samples: control mousse (F0), mousse fortified via direct addition (F1), mousse fortified via encapsulation (F2), potato starch, and pure grape seed oil.

Fourier transform infrared spectroscopy in attenuated total reflectance mode (FTIR-ATR) was employed to characterize the molecular interactions and structural changes in the formulated fruit mousses. The spectra (Figure 2) of grape seed oil and potato starch were analyzed as references, followed by a comparative assessment of the control (F0) and experimental samples (F1 and F2). This approach aligns with established protocols for monitoring lipid oxidation and encapsulation efficiency in complex food matrices without prior fat extraction [32,33].

The spectrum of grape seed oil exhibited characteristic bands at ~2926 cm^−1^ (asymmetric CH_2_ stretching), ~1740 cm^−1^ (C=O ester stretching of triglycerides), and 1640–1650 cm^−1^ (C=C stretching of unsaturated bonds). The carbonyl band at 1740 cm^−1^ served as a key indicator for triglyceride integrity. Potato starch showed a broad O–H stretching band (3000–3600 cm^−1^), a peak near 2927 cm^−1^ (C–H stretching), and a distinct region between 1000 and 1150 cm^−1^ (C–O–C glycosidic linkages), characteristic of its polysaccharide structures [34,35,36,37].

The F0 sample displayed dominant carbohydrate and moisture-related bands, with no detectable lipid signals. In F1 (mousse supplemented with free oil, starch, and plant extracts), weak lipid bands at ~1740 cm^−1^ and ~2926 cm^−1^ were detectable, but overlapped with matrix absorptions, indicating the presence of unencapsulated oil. In contrast, the spectrum of F2 (mousse with starch-encapsulated nanocarriers) revealed a marked increase in intensity at ~1740 cm^−1^ relative to F1, signifying a higher effective concentration of ester carbonyl groups due to oil stabilization with the starch matrix. Concurrent modifications in the 1000–1150 cm^−1^ region suggested structural reorganization of starch glycosidic bonds resulting from starch–lipid interactions. The improved resolution of the 2926 cm^−1^ band in F2 indicated a more homogeneous lipid dispersion and reduced spectral overlap with the polysaccharide matrix, consistent with effective oil–starch association [32,36,38]. Additionally, the reduction in the prominent water-associated band at ~1640 cm^−1^ in F2 suggested decreased water accessibility to the oil phase, supporting successful encapsulation and enhanced oxidative stability [32,36,39,40].

#### 2.1.3. Water Content, Water Activity, and pH of Mousses

Table 1 summarizes the water content, water activity (a_w_), and pH values of the analyzed fruit mousses.

The fortified mousses (F1 and F2) did not show significant changes in moisture content; however, slight variations in water activity were observed among the samples. The a_w_ values for all mousses ranged from 0.75 to 0.84. The pH values of the mousses were within the typical range for this type of fruit preserve (~3.6–3.8) [41,42,43]. Fortification resulted in a slight increase in pH; however, the change was minimal and not biologically significant. From a technological standpoint, the pH remained within a safe range, thereby supporting product stability and shelf life. It is well established that water activity, temperature, and pH interact to determine the conditions that inhibit microbial growth [44]. Considering these three parameters together, it can be concluded that the measured values in the mousses are sufficient to effectively inhibit the growth of most pathogenic and spoilage microorganisms, including *Clostridium botulinum*, *Salmonella* spp., *Staphylococcus aureus*, *Listeria monocytogenes*, and *Escherichia coli*. However, it should be noted that osmophilic yeasts and xerophilic molds may still pose a potential risk due to their broad tolerance to low pH, reduced temperatures, and limited water activity [45,46]. Most microorganisms grow optimally at neutral pH, although some species are capable of developing in acidic environments depending on temperature and water activity (a_w_). For example, the optimal conditions for the growth of *Staphylococcus aureus* range from 30 to 37 °C and pH 6.0–7.0; however, this pathogen can also proliferate under less favorable conditions, tolerating temperatures between 7 and 48.5 °C and pH values from 4 to 10 [47]. Therefore, ensuring adequate protection of the product against microbial spoilage is essential. Refrigerated storage of the mousses (approximately 6 °C) helps to limit the growth of mesophilic bacteria [48] and suppresses the development of pathogens with broader environmental tolerance [49]. Additionally, the fruit mousse production process included pasteurization, which enhances product safety by eliminating vegetative bacterial cells and reducing enzymatic activity [50].

#### 2.1.4. Color Parameters of Mousses

Table 2 presents the parameters characterizing the color of the fruit mousses. In colorimetry, color is described by three parameters: hue angle (h*), chroma (C*), and lightness (L*).

The analyzed mousses exhibited moderate lightness values, averaging approximately 55, which is consistent with the values reported for banana puree by Moniharapon et al. [51] but higher than those reported for strawberry purees by Teriba [52] and Kim [53]. All samples showed a predominance of red and yellow tones, as indicated by the positive a* and b* coordinates [29]. Compared with the control sample (F0), the mousses enriched with bioactive substances exhibited higher color saturation, which was directly associated with increased C values, reflecting greater color intensity. In the present study, the h* values, which define the qualitative aspect of color, corresponded to a light red hue. The addition of elderberry extract and grape seed oil resulted in only a slight increase in brightness. Considering the strong coloring properties of elderberry extract, a more pronounced darkening of the product was expected. However, the mousse formulations also contained strawberries, which impact the red hue, and bananas, which are prone to enzymatic browning upon exposure to oxygen and light. Therefore, it can be concluded that the incorporation of bioactive compounds, regardless of whether they were delivered via encapsulation (F2) or direct addition (F1), did not result in perceptible darkening of the product.

The color attributes of fruit-based products such as mousses, pulps, or purees are largely dependent on the type and composition of raw materials used. However, it should be emphasized that many fruits are susceptible to color changes resulting from mechanical and thermal processing, as well as environmental exposure. Naturally occurring pigment degradation often leads to undesirable brown discoloration, which is generally unacceptable to consumers [54,55]. Color is one of the most important attributes influencing food quality and consumer perception. In addition, it can act as a useful indicator of chemical and microbiological alterations taking place in food [55,56]. In strawberries, color deterioration is predominantly associated with the degradation of anthocyanin pigments responsible for their characteristic vivid red hue, with pelargonidin-3-glucoside and cyanidin-3-glucoside identified as the major contributors [57,58,59]. Browning of apple and banana pulp is mainly attributed to enzymatic reactions, in which oxidative enzymes—polyphenol oxidase (PPO) and peroxidase (POD)—catalyze the oxidation of phenolic compounds (e.g., chlorogenic acid, catechins) [60,61,62].

Based on the chromatic and lightness measurements, the total color difference (ΔE*) between the enriched samples (F1, F2) and the control (F0) was calculated, yielding values ranging from 0.53 to 0.97. Products with ΔE* values below 1, such as F1 and F2, are considered to exhibit a very high degree of visual similarity to the control [63,64,65]. Consequently, for the average observer, the differences between F0 and F1/F2 are imperceptible to the naked eye. From an industrial perspective, this finding suggests that such additives may be incorporated without causing noticeable changes in product appearance, which is advantageous when preservation of a characteristic visual profile is desired. According to widely accepted thresholds, a ΔE* value of at least 3.5 is required for a color change to be clearly perceptible to the average consumer [64].

#### 2.1.5. Rheological Characteristics of Mousses

**Figure 3:**
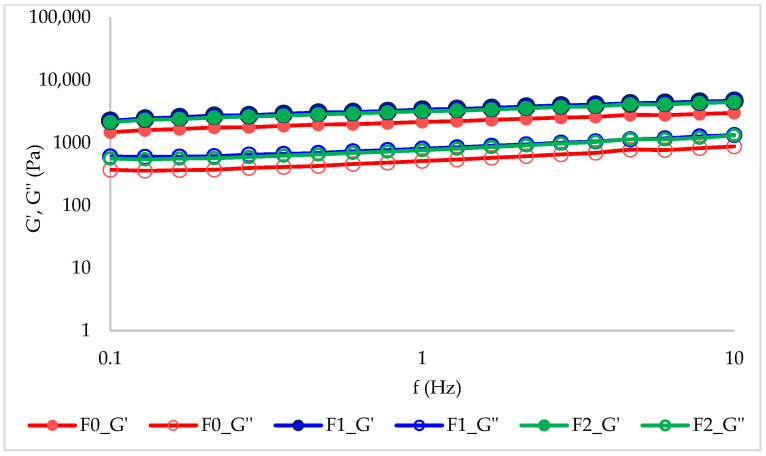
Frequency-dependent behavior of mechanical moduli (G′ and G″) in fruit mousse samples.

Figure 3 displays the storage modulus (G′) and loss modulus (G″) as functions of oscillation frequency (f) for the examined fruit and vegetable dessert samples.

All mousses exhibited a gel-like behavior, as the storage modulus exceeded the loss modulus (G′ > G″) across the entire tested frequency range (0.1–10 Hz). Both moduli increased with frequency; however, the increase was slight, indicating weak frequency dependence and suggesting a solid-like response of the dispersions [66]. The control sample (F0) exhibited lower mechanical moduli compared with the other samples, which showed comparable G′ and G″ values, indicating a stiffer yet more elastic structural network. Fruit purees are concentrated dispersions consisting of soft, deformable, and insoluble particles suspended in an aqueous phase. This liquid phase contains sugars, organic acids, and pectic compounds [67]. The rheological properties of fruit mousses are governed by these two phases. For the insoluble fraction, key determining factors include particle shape, rigidity, and size distribution. An increase in particle content and particle size enhances viscoelastic properties, whereas particle size reduction and the addition of pectin have the opposite effect [68]. The ripeness stage of bananas also influences the rheological properties of the mousse. In unripe bananas, the starch content may reach up to 74%, whereas in ripe bananas, due to starch hydrolysis, this value decreases drastically to around 1%. At the same time, the content of simple sugars increases [69]. Therefore, careful control of raw material maturity is essential during mousse production. Additional factors influencing rheological behavior include dry matter content and the pH of the system. A higher water content generally results in lower values of both mechanical moduli. The pH of the fruit, in turn, affects the conformation of pectins and proteins as well as electrostatic interactions. According to Asimakopoulou et al. [70], at pH 3, citrus pectin exhibits shear-thinning behavior. In contrast, at pH 7, higher viscosity and more rigid chain behavior are observed, resulting from electrostatic repulsion between ionized acidic groups.

The incorporation of bioactive substances into the mousses (F1 and F2) enhanced their viscoelastic properties. The applied encapsulation technique enabled effective homogenization of lipid droplets, while the presence of starch gel and naturally occurring pectins in the raw material helped stabilize the emulsions. Through their hydrophobic groups, these components reduced the interfacial tension to a level sufficient to maintain system stability. It should be noted, however, that native starch, which forms a loosely packed adsorption layer at the oil–water interface, exhibits a weaker ability to reduce interfacial tension compared with modified starches (e.g., acetylated or cross-linked starches) [71].

Our previous studies have confirmed the positive effect of the encapsulation process on the rheological properties of food products, which in turn translates into improved sensory attributes. Specifically, in our analysis of the impact of encapsulated walnut oil on the rheological characteristics of yoghurt, we demonstrated that its incorporation increased yoghurt’s viscosity and enhanced its storage stability compared with the control sample without capsules [17]. Similarly, other studies [4] have reported that encapsulation partially mitigates the adverse effects associated with the addition of lipids and propolis, indicating improved structural integrity resulting from controlled release of the oil phase.

#### 2.1.6. Textural Parameters of Mousses

The parameters of the fruit mousses are presented in Table 3. Texture analysis was performed on the fresh product.

As shown by the tabulated data, the hardness values of the mousses were low, indicating that only a small force was required to disrupt their structure. Fruit purees and pulps generally lose the mechanical properties of the original raw materials due to the rupture of plant cell walls and the dissolution of pectic substances. These textural changes can also influence consumer perception.

The final texture of a pulp is influenced by numerous factors, but is primarily determined by the type of plant material used. In this study, the raw materials consist mainly of soft fruits, namely strawberries and bananas, with apples being the only firm component. Fruits are a source of dietary fiber, predominantly the soluble fraction, which is mainly represented by pectins [72,73].

The hardness of fruit pulps is also affected by water content and applied technological processing. For fruit mousses intended for use as toppings or snacks, it is important that their textural properties enable easy dispensing while maintaining a smooth and delicate structure. Excessive adhesiveness, manifested as stickiness, tackiness, or stringiness, may negatively affect consumer acceptance. Products with low adhesiveness are perceived as light, delicate, and refreshing.

In the case of the analyzed fruit mousses, enrichment with bioactive substances (F1 and F2) did not result in significant changes to the textural parameters, which may positively influence the sensory quality and supports the effectiveness of the applied fortification strategy.

#### 2.1.7. Sensory Parameters of Mousses

**Figure 4:**
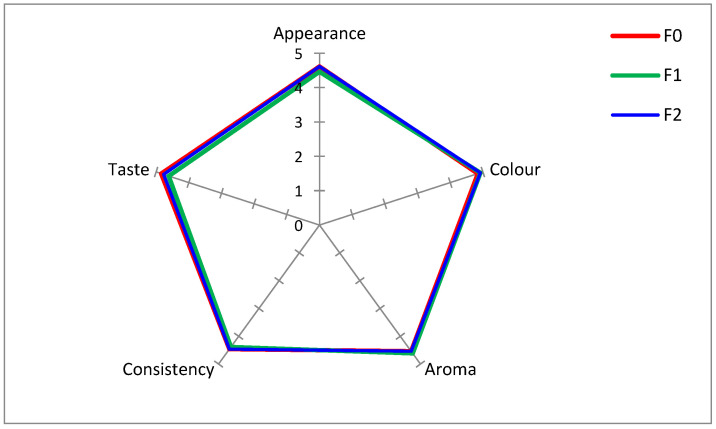
Quality factors of mousses. Statistical differences in quality attributes between formulations were evaluated using one-way ANOVA followed by Fisher’s post hoc test (*p* < 0.05). No statistically significant differences were observed.

**Figure 5:**
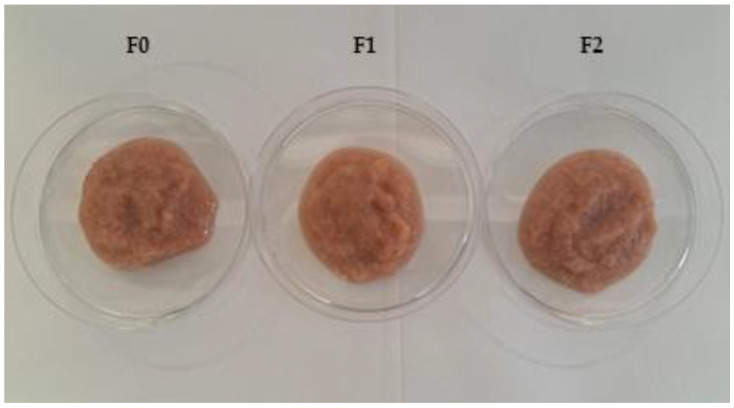
Photographs of developed mousses.

The high sensory scores confirm that the use of starch gel as a neutral additive does not negatively affect the sensory perception of the desserts (F0) (Figure 4 and Figure 5). Sample F1, exhibited slightly lower ratings (but statistically insignificant) for consistency and taste, which may be attributed to the presence of lipid components and elderberry extract, potentially altering the product. Nevertheless, attributes such as appearance, color, and aroma remained at a high level, comparable to the control sample, indicating strong consumer acceptance of this type of fortification.

In the sample containing the nanoemulsion (F2), no differences were observed in aroma or consistency, demonstrating effective masking of the added oil and extract. The overall acceptance of the fortified mousses ranged from 4.5 to 4.7.

Similar observations have been reported in previous studies on food fortification with bioactive compounds. Several authors have demonstrated that the direct addition of plant extracts or lipid fractions may adversely affect aroma and texture, primarily due to the presence of volatile compounds and disturbances in the fat–water balance of the system, which can lead to the perception of an undesirable oily aftertaste by consumers. However, it has been shown that the use of polysaccharide-based encapsulation systems effectively masks undesirable sensory properties, in particular bitterness, undesirable taste, and texture defects associated with lipid fortification [4,17,74]. For example, studies on apple–plum mousses and yoghurts enriched with encapsulated bioactive compound or oils showed no significant differences in aroma, texture, or overall acceptability compared to control samples, provided that the droplet size was sufficiently reduced and a compatible stabilizing matrix was used [4,19].

#### 2.1.8. Polyphenol Content and Antioxidant Activity

The tested mousses exhibited very similar values of total polyphenol content (TPC). The obtained TPC values, expressed as gallic acid equivalents (mg GAE) per 100 g of product, ranged from 73.16 (for mousse F0) to 78.64 (for mousse F2). A slightly lower variation, however, not statistically significant (*p* > 0.05) was observed in the case of total flavonoid content (TFC), determined using the reaction with aluminum chloride (AlCl_3_); these values ranged from 1.088 (for mousse F0) to 1.090 (for mousse F1) and were expressed as quercetin equivalents (mg QE) per 100 g of mousse (Table 4).

The analyzed mousses exhibited notable antioxidant activity, assessed using the ABTS and DPPH radical scavenging assays. The tested extracts demonstrated differentiated statistically (*p* < 0.05) antiradical activity against a DPPH radical, ranging from 0.3670 (for mousse F0) to 0.4130 mM TE/100 g (for mousse F2) and were expressed as Trolox equivalents (TE). A high linear correlation coefficient was observed between the result obtained from the DPPH assay and that of TPC (r = 0.9968). This fact indicates that polyphenolic compounds determined using the Folin method play the main role in shaping the antiradical activity of the tested extracts.

In the case of the ABTS assay, it was found that the extracts, similarly to the DPPH method applied, showed significant antioxidant activity, ranging from 1.0579 to 1.1877 mM TE/100 g of mousse. The antiradical activity against that cation radical of mousses F0 and F1 was very similar, whereas a slightly and significantly (*p* < 0.05) higher activity against this radical was observed for the extract obtained from mousse F2 (1.1877 mM/100 g).

A high linear correlation was observed between the ABTS assay and the Folin method (r = 0.9008), confirming the significant contribution of phenolic compounds to their antiradical activity against ABTS^•+^. An important property of the analyzed extracts is their reducing activity toward Fe^+3^ ions, demonstrated using the FRAP assay (ferric ion reducing antioxidant parameter). It was found that the extracts showed a high ferric ion (Fe^+3^)-reducing capacity, ranging from 0.9886 (for mousse F0) to 1.0558 (for mousse F2) and were expressed as mM of Fe^+3^ ions reduced to ferrous ions (Fe^2+^) by the extract amount corresponding to 100 g of mousse. The reducing activity of mousse F2 was significantly higher (*p* < 0.05) when compared with the remaining samples. Strong linear correlations were observed between the FRAP and TPC values (r = 0.9981), as well as the DPPH assay (r = 0.9899). This indicates that the determined phenolic compounds are responsible for the antiradical activity of products and their reducing capacity.

When comparing total flavonoid content (TFC) in the tested water–methanol extracts, it was observed that the values obtained using that method were somewhat underestimated relative to those determined chromatographically (i.e., the content of phenolic acids and flavonoids) (Table 5). This discrepancy is likely associated with the relatively low specificity of that spectrophotometric method when compare with chromatographic method, in addition to using quercetin as the reference standard, since that flavonoid is the most commonly applied standard for this purpose. Quercetin belongs to the flavonol subclass of flavonoids, whereas the only flavonoids identified chromatographically in the studied samples, i.e., catechin and hesperidin, belong to the other two flavonoid subclasses (flavanols and flavanones). Notable structural differences exist between these groups, including variations in the spatial orientation of their molecular fragments and the number of hydroxyl groups. Such differences may modulate their reactivity toward aluminum chloride, which forms colored complexes with flavonoids, thereby contributing to variation in spectrophotometric quantification.

In the analyzed mousses, the phenolic compounds belonging to phenolic acids and flavonoids were identified. Among the phenolic acids, gallic acid (the only hydroxybenzoic acid) and two hydroxycinnamic acids, including *p*-coumaric and caffeic acids, were found. Two flavonoids were also identified by means of HPLC: (+)-catechin (flavanol) and hesperidin (flavanone). In extracts obtained from the samples not subjected to hydrolysis, only two phenolic compounds were present in free form: gallic acid and catechin. The gallic acid content was very similar across the mousses studied, ranging from 0.2904 mg/100 g (mousse F2) to 0.3056 mg/100 g (mousse F0). On the contrary, catechin was present at considerably higher levels and showed greater variability, ranging from 4.44 mg/100 g (mousse F0) to 7.18 mg/100 g (mousse F2). Catechin itself, being a member of the flavanol subgroup of flavonoids, naturally occurs in plant products only in its free form (aglycone), which explains its presence in the analyzed water–methanol extracts that were not subjected to alkaline hydrolysis.

As a result of alkaline hydrolysis of the water–methanol extracts, the part of phenolic compounds usually present in raw products as bound forms (i.e., as organic acid esters or glycosides) was released. This allowed for the identification of hydroxycinnamic acids (i.e., caffeic and *p*-coumaric acids) as well as hesperidin, which naturally occurs in plant materials predominantly in the bound form (typically as 7-O-glycosides). Caffeic acid content in the analyzed mousses was similar across the samples, ranging from 0.88 mg/100 g (mousse F0) to 1.07 mg/100 g (mousse F2). However, a noticeable difference was observed between the control mousse (F0) and the remaining samples. *p*-Coumaric acid was present in somewhat lower quantities, ranging from 0.537 to 0.592 mg/100 g, with the lowest value detected in mousse F0 and the highest in mousse F2. Hesperidin content showed markedly greater variability among samples compared with phenolic acids, ranging from 0.2799 mg/100 g (F0 control mousse) to 0.4367 mg/100 g (mousse F2). The obtained amounts for that flavonoid differed statistically significantly (*p* < 0.05) among all of the studied mousses. The total content of both phenolic acids and flavonoids determined chromatographically was significantly lower than that of the TPC measured using the Folin method. This discrepancy arises from the relatively low specificity of the Folin reagent, which besides polyphenols themselves may also react with a range of reducing substances, including sugars, ascorbic acid, aromatic amines, sulfur dioxide, aldehydes, proteins, ferrous ions, urea, and others, leading to inflation (overestimation) of TPC values when using only the Folin method for comparison [75].

Regardless of these observations, high linear correlation coefficients were found between the DPPH assay and the contents of phenolic compounds determined by means of HPLC, including caffeic (r = 0.9492) and *p*-coumaric acids (r = 0.9457), and the contents of flavonoids, such as catechin (r = 0.9465) and hesperetin (r = 0.9611). These findings indicate that such compounds play an important role in determining the DPPH radical scavenging activity of the analyzed extracts. Such correlations were not observed when comparing the values of certain phenolic compounds with antioxidant activity in the extracts estimated using the ABTS assay.

High linear correlation coefficients were also observed between the FRAP values of the extracts and the contents of caffeic (r = 0.9839), *p*-coumaric acid (r = 0.9819), and flavonoids including catechin (r = 0.9819) and hesperetin (r = 0.9903). These findings indicate that the above-mentioned phenolics play an important role in determining the reducing activity of the tested extracts.

Although the TPC and TFC were comparable in both fortified samples (F1 and F2), significantly higher antioxidant activity (i.e., DPPH, ABTS assays) and higher contents of selected phenolic compounds (Table 5) were observed in the sample enriched with the encapsulated extract (F2). This suggests that the encapsulation effectively protected sensitive bioactive compounds against degradation during thermal processing, such as pasteurization, preserving their functional antioxidant properties. This was reflected in the highest content of certain phenolic compounds (Table 5), such as catechin and hesperidin, as well as *p*-coumaric and caffeic acids, with the highest amounts recorded for encapsulated mousse F2.

#### 2.1.9. Microbiological Parameters of Mousses

- Microbiological stability during storage

Examining microbiological purity during storage of the fruit mousse samples revealed excellent microbiological stability throughout the observation period. Across the sampling intervals, no microbial growth was detected on selective media and only two bacterial colonies were observed on Trypticase Soya agar from a single sample at the first time point. This minimal growth suggests that the mousses maintained a high level of microbiological purity and were not prone to contamination under the applied storage conditions. The absence of microbial proliferation can be attributed to low water activity and an acidic pH, typical of fruit-based products, which inhibit the growth of most spoilage and pathogenic microorganisms [76], or the presence of natural antimicrobial compounds in fruit ingredients, such as organic acids and phenolic compounds, which exert bacteriostatic or even bactericidal effects [77]. Importantly, proper storage conditions (refrigeration and hygienic packaging) will further limit microbial development. These findings align with previous reports indicating that fruit-based products, when processed and stored under controlled conditions, exhibit extended microbiological shelf life [78,79]. The detection of only two bacterial colonies on general medium suggests incidental contamination rather than systemic spoilage, and the observed count remained far below levels considered hazardous for consumer health. From a food safety perspective, the results confirm that the tested mousses meet microbiological criteria for ready-to-eat fruit products during the evaluated storage period. This stability provides a reliable baseline for subsequent evaluation of functional properties, such as the antibacterial activity of incorporated elderberry extracts. At the same time, it should be noted that the absence of microbial growth indicates concentrations below the detection limit of the applied methods (1 CFU/g) rather than confirmed absolute sterility of the products.

- Antibacterial activity of products

**Figure 6:**
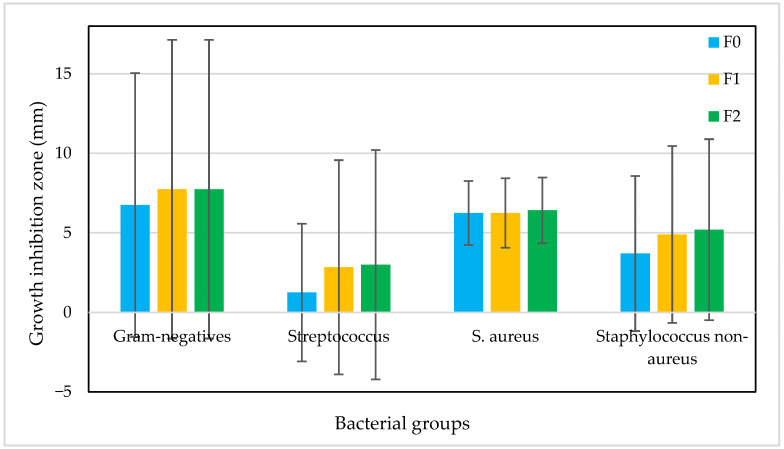
Growth inhibition zones (mm) of selected bacterial groups determined for control (F0) and fortified fruit mousses (F1 and F2). Bars represent mean ± SD. Statistical significance of differences in antimicrobial activity between formulations was assessed using ANOVA with Tukey post hoc test (*p* < 0.05). Only F0 activity differed from other two formulations (Gram-negatives vs. *Streptococcus* spp.; F = 3.08, *p* = 0.04).

**Figure 7:**
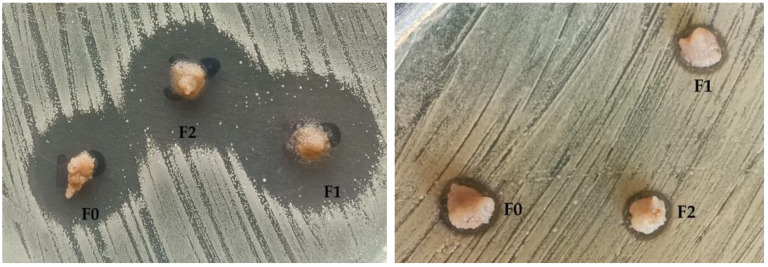
Photos showing the growth inhibition of bacterial cultures caused by the fruit mousses. On the left—*Moraxella catarrhalis*; on the right—*Staphylococcus aureus*.

The antibacterial activity of the fruit mousses (F0, F1, and F2) was examined against 38 Gram-positive and Gram-negative bacteria using a surface application assay. The absence or presence (and the size if present) of growth inhibition zones around the applied mousses was recorded. The results demonstrated differences in antibacterial activity between mousse variants and bacterial groups and species (Figure 6 and Figure 7).

In general, there were far less Gram-negative bacteria examined (i.e., only four strains) compared to Gram-positives (i.e., *n* = 34). Among Gram-negatives, *Klebsiella pneumoniae* and *Stenotrophomonas maltophilia* exhibited no inhibition (0 mm) for all mousse variants, suggesting limited efficacy against these species. The other two, *Moraxella catarrhalis* and *Neisseria flavescens*, showed much larger growth inhibition zones (i.e., 17–19 and 10–12, respectively). Among Gram-positive bacteria, *Streptococcus mitis* showed the largest growth inhibition zones (15–22), while the majority of other *Streptococcus* spp. strains showed no reaction, suggesting selective activity of the compounds contained in the mousses. The most even growth inhibition was observed in the case of *S. aureus* (6–8 mm in the majority of strains). Figure 6 shows the mean values of the growth inhibition zones (mm) with standard deviations, while Figure 7 presents selected photos showing the growth inhibition of bacterial cultures caused by the fruit mousses. Importantly, the differences in growth inhibition were statistically significant in the case of F0 (Gram-negatives vs. *Streptococcus* spp.), whereas other differences (i.e., between other groups of bacteria and between mousse variants) were not significant (*p* > 0.05). When comparing the mean values, F1 and F2 generally produced larger growth inhibition zones, indicating that the addition of the elderberry extract slightly enhanced antibacterial activity. However, these differences were minor in the majority of cases and could be noticed only in the case of three strains, i.e., *Streptococcus mitis* (15, 20, and 22 mm for F0, F1, and F2, respectively) and two *Staphylococcus* spp. strains (0, 10, and 10 and 11, 15, and 14 for F0, F1, and F2). The evenness of the bacterial reaction to the three mousse types indicates that the elderberry extract was not the most important factor influencing the antibacterial effects of the mousses in vitro. For this reason, we assume that the antibacterial effect observed in our study could be primarily attributed to the content of polyphenols and other phenolic compounds. Such an effect has been described and discussed by Ribeiro et al. [80] for apple fruit, Barkaoui et al. [81] for strawberries, and Kanedi et al. [82] for bananas. Nevertheless, the bioactive potential, including antimicrobial activity, of elderberry and its extracts has also been described in the literature [79,83].

## 3. Materials and Methods

### 3.1. Materials

Base ingredients of the fruit mousse: strawberries (*Fragaria × ananassa*), ripe bananas (*Musa acuminata*), and apple (*Malus domestica*) (Gala). Dry elderberry fruit (*Sambucus nigra* L.) purchased from DARY NATURY Sp. z o.o. (Koryciny, Poland) was used to obtain the elderberry extract. Potato starch (PS) [Superior Standard] was purchased from PPZ Bronisław (Strzelno, Poland). Ethyl alcohol 96% p.a. grade was purchased from F.H.U. DOR-CHEM (Cracow, Poland) and used for obtaining the elderberry extract. Grape seed oil from Monini was purchased in a neighborhood store.

### 3.2. Extraction of Elderberry Fruit

The dried elderberry fruits were ground into a fine powder using an RCMZ-800N grinder (Royal Catering, Berlin, Germany). A 10.0 g portion of the powdered elderberry fruits was mixed with 100 mL of ethanol (96% p.a. grade). The extract was obtained via continuous ethanol extraction using a Soxhlet apparatus for 6 h. After extraction, the solvent was removed under reduced pressure using a rotary evaporator (Heidolph instruments GmbH&Co. KG, Schwabach, Germany) at 40 °C, yielding a dry extract.

### 3.3. Preparation of Starch Gel

A 4% (*w*/*w*, dry matter) potato starch gel was prepared by dispersing starch in distilled water and heating the mixture at 95 °C for 1 h under continuous stirring (700 rpm). After heating, the gel was cooled and further stirred at 23 °C for an additional 1 h.

### 3.4. Preparation of Nanoemulsion

To prepare the nanoemulsion, dry elderberry fruit extract, grape seed oil, and distilled water were mixed according to the proportions shown in Table 6. The encapsulation conditions were optimized in preliminary experiments. The mixture was homogenized using an ultrasonic homogenizer (Bandelin Electronic GmbH & Co. KG, Berlin, Germany) (at 40% amplitude, approximately 20 W output power) for 5 min in continuous mode under cooling conditions (2–4 °C) using an ice–water bath. Cooling was applied to prevent temperature increase, which could otherwise lead to bioactive compound degradation and oil oxidation. Ultrasonic homogenization was performed to reduce oil droplet size and obtain a nanoemulsion. Subsequently, potato starch gel (prepared according to Section 3.3) was added dropwise using an automatic pipette to stabilize the nanoemulsion by forming a protective coating around the droplets. In the final step, the prepared nanoemulsion was incorporated into the fruit mousse matrix.

### 3.5. Mousses Preparation

Fruits (strawberry, bananas, apple) were peeled, cut into small pieces, and then blended into a homogeneous mixture using a kitchen blender (Götze & Jensen, Copenhagen, Denmark) until a uniform puree was obtained. They were then cooked for 10 min. The base fruit mousse was enriched with additional ingredients (Table 6), depending on the mousse variant:

F0—fruit mousse with the addition of potato starch gel (control sample).

F1—fruit mousse enriched via direct fortification with the addition of starch gel, elderberry extract, grape seed oil, and distilled water.

F2—fruit mousse enriched with a nanoemulsion (obtained in accordance with the description in Section 3.4).

After thoroughly mixing all the ingredients, the product was transferred into small glass jars, which were then pasteurized in hot water for 20 min. Once cooled, the samples were stored under refrigerated conditions at 6 °C.

### 3.6. Physicochemical Analysis

#### 3.6.1. Sample Preparation

For further examinations, 70 g portions of each mousse formulation were dried at 40 °C for roughly 24 h. These dehydrated samples served as the material for Scanning Electron Microscopy (SEM) and Fourier transform infrared spectroscopy (FTIR) analyses. In turn, color assessment, texture profiling, rheological testing, and sensory analysis were carried out using the freshly prepared mousses.

#### 3.6.2. Scanning Electron Microscopy (SEM)

The surface structure of the dried, encapsulated mousse was analyzed with a JEOL 7550 scanning electron microscope (JEOL Ltd., Akishima, Tokyo, Japan). Prior to imaging, the sample was coated with a 20 nm chromium layer using a K575X Turbo Sputter Coater (Emitech Ltd., Kent, UK) to ensure adequate electrical conductivity.

#### 3.6.3. Fourier Transform Infrared Spectroscopy (FTIR)

Samples of each variant of the dried fruit mousse, as well as grape seed oil and potato starch, were used for the analysis. FTIR spectra were obtained using a MATTSON 3000 FT-IR spectrophotometer (Madison, WI, USA) equipped with a 30SPEC 30 Degree Reflectance accessory (MIRacle ATR, PIKE Technologies Inc., Madison, WI, USA). Measurements were carried out at 25 °C (±2 °C), and the spectra were recorded within the wavenumber range of 4000–700 cm^−1^.

#### 3.6.4. Moisture Content

The moisture content of the samples was determined using the drying oven method. For this purpose, 1 g of mousse was placed in a MAC 50 moisture analyzer (Radwag, Radom, Poland) and dried until a constant weight was achieved. Each measurement was performed in duplicate for each mousse variant.

#### 3.6.5. Water Activity

Water activity was measured using an AquaLab 4TE device (Decagon Devices, Inc., Pullman, WA, USA), in accordance with ISO 18787:2017 [84]. Measurements were performed at a temperature of 20 °C (293 K) in duplicate for each sample after temperature equilibration.

#### 3.6.6. Determination of pH

The pH of the mousses was determined using a pH meter, PH-100 ATC (Voltcraft, Hirschau, Germany), measuring the electromotive force, which was used to calculate the potential of the sample.

#### 3.6.7. Color Measurement

The color of the mousses was assessed using a Konica Minolta CM-3500d spectrophotometer (Konica Minolta Inc., Tokyo, Japan) equipped with a 30 mm measurement aperture, operating under D65 illumination and a 10° standard observer. Color parameters were determined in the CIELAB system, including L*, a*, and b*. The L* coordinate indicates lightness, ranging from 0 (black) to 100 (white). The a* coordinate represents the green–red axis, with negative values corresponding to green and positive values to red. Likewise, the b* coordinate denotes the blue–yellow axis, where negative values indicate blue and positive values indicate yellow.

Each sample was measured five times, and the mean value was used for analysis. Based on the obtained data, additional color attributes—chroma (C*), hue angle (h*), and total color difference (∆E*)—were also calculated [4].

Saturation (chroma, C*) represents the intensity or purity of a color relative to a neutral gray of the same lightness. Higher C* values correspond to more vivid and visually striking colors.

(1) $$C^{*}=\sqrt{{a*}^{2}+{b*}^{2}}$$

The hue angle (h*) indicates the position of a color on the three-dimensional color wheel. Common reference points include 0° for red, 90° for yellow, 180° for green, and 270° for blue.

(2) $$h*={tan}^{-1}\left(\frac{b*}{a*}\right)$$

The total color difference (∆E*) expresses the extent of color variation between a given sample and the reference (control) sample [85].

$${∆E}^{*}=\sqrt{{∆a*}^{2}+{∆b*}^{2}+∆{L*}^{2}}$$

#### 3.6.8. Rheological Measurements

The rheological behavior of the samples was examined following the procedure outlined by Krystyjan et al. [30], with minor adjustments. A RheoStress RS 6000 rotational rheometer (Thermo Scientific, Karlsruhe, Germany) equipped with a P35Ti plate–plate geometry was used for the analyses. The temperature of the lower plate was kept constant at 22.0 ± 0.1 °C. All measurements were performed on freshly prepared mousses, and each test was repeated three times.

During the frequency sweep test, the oscillation frequency was varied from 0.01 to 30 Hz at a constant deformation of 1 Pa, ensuring that the measurements were conducted within the linear viscoelastic region.

#### 3.6.9. Texture Analysis

The textural characteristics of the fruit mousses were evaluated using a TA.XTplus texture analyser (Stable Micro Systems Ltd., Godalming, UK) according to Krystyjan et al., with some modifications [4]. A Texture Profile Analysis (TPA) test was conducted with a cylindrical probe (20 mm diameter), operating at a speed of 1 mm/s. The probe performed double compression to a depth of 25 mm under a constant load of 10 g. Samples were placed in jars with a diameter of 55 mm, and measurements were carried out at 22.0 ± 0.1 °C. Force–distance curves generated during the test were used to calculate key textural parameters, including hardness (N), adhesiveness (N·s), cohesiveness (dimensionless), and chewiness (N).

#### 3.6.10. Sensory Analysis

The sensory evaluation was conducted by a trained sensory panel consisting of 11 assessors (*n* = 11). Panelists were recruited and trained in accordance with the relevant ISO standards (ISO 3972:2011, ISO 5492:2008, ISO 8586:2023, ISO 11132:2021, and ISO 6658:2017) [86,87,88,89,90]. All analyses were conducted in a sensory laboratory designed in compliance with PN-EN ISO 8589:2007 [91]. The study protocol was approved by the University Ethics Committee for Research Involving Humans (approval number 330/2025). Sensory evaluation was carried out using a five-point scaling method in accordance with ISO 4121:2003 [92]. The mousses were evaluated immediately after preparation. A weighted scoring system was applied with the following quality attributes and weighting coefficients: appearance (0.10), color (0.15), aroma (0.20), consistency (0.25), and taste (0.30). Overall acceptance was calculated as the weighted sum of individual attributes. Scores < 2.9 were considered unacceptable, 3.0–3.5 acceptable, 3.51–4.5 good, and 4.51–5.0 very good, according to Krystyjan et al. [93].

#### 3.6.11. Polyphenols and Antioxidant Activity

- The procedure for the extraction of phenolic compounds from the analyzed fruit mousses

In order to extract phenolic compounds from the analyzed fruit mousses, a polyphenol phenolic extraction procedure was established using aqueous–methanol solutions of different concentrations and deionized water. The extraction efficiency of polyphenols in the obtained extracts was then evaluated using the Folin–Ciocalteu method. It was found that the extract obtained using an 80:20 (*v*/*v*) methanol–water mixture exhibited the highest polyphenol extraction efficiency. For the extraction of phenolic compounds from the tested mousses, approximately 10 g of each sample was weighed and transferred into a 100 mL volumetric flask. The flask was then filled with a methanol–water mixture (80:20, *v*/*v*) to a volume of approximately 60 mL of extraction solvent. The resulting mixtures were covered with aluminum foil to protect them from light and extracted on a mechanical shaker at room temperature for 24 h. After this period, the contents of the flask were made up to 100 mL with the extraction mixture. The resulting extracts were filtered and subsequently defatted in a separatory funnel through triple extraction with petroleum ether, using a petroleum ether-to-sample volume ratio of 1:3.

The obtained defatted aqueous–methanolic extracts were subsequently subjected to chromatographic analysis. For this purpose, the extracts were preliminarily purified using solid-phase extraction (SPE). An SPE cartridge (Hypersil–Keystone, Thermo Scientific, Waltham, MA, USA) packed with RP-18 sorbent (1 g filling mass, 6 mL column volume) was first conditioned with 5 mL of methanol. Then, 3 mL of the analyzed extract was applied to the cartridge and eluted into a 10 mL volumetric flask, followed by methanol to make up the volume to 10 mL. The resulting purified extract was directly subjected to chromatographic or spectrophotometric analysis.

- The determination of total phenolic content in the analyzed fruit mousses using a spectrophotometric method

Total phenolic content (TPC) in the analyzed samples was determined using the Folin–Ciocalteu method developed by Singleton and Rossi [94], based on the aqueous–methanolic extracts obtained from these products. For this purpose, 0.5 mL of the extract was mixed in a test tube with 2.5 mL of Folin–Ciocalteu reagent previously diluted tenfold with deionized water. After 5 min of incubation, 2 mL of a 7.5% (*m*/*v*) sodium carbonate solution was added to each tube. A blank sample was prepared analogously, using deionized water instead of the extract. After 2 h, the absorbance of the resulting solutions was measured using a UV/Vis spectrophotometer (Jasco, Tokyo, Japan) against the blank sample. The results were expressed as milligrams of gallic acid equivalents (GAE) per 100 g of mousse, calculated as the arithmetic mean of three analytical replicates. The calculations were based on the calibration curve equation y = 0.0127x + 0.0168, where y represents the absorbance of the tested sample and x represents the gallic acid concentration in mg/L.

- The determination of total flavonoid content in the tested fruit mousses using a spectrophotometric method

Total flavonoid content in the analyzed samples was determined using a spectrophotometric method developed by Barnum et al. [95], based on the water–methanol extracts obtained from these products. For this purpose, 1 mL of the tested water–methanol extract was mixed in a test tube with 4 mL of deionized water. Subsequently, 0.3 mL of a 15% (*m*/*v*) aqueous sodium nitrite (NaNO_2_) solution, 0.3 mL of a 10% (*m*/*v*) aluminum chloride solution in methanol, and finally 4 mL of a 4% (*m*/*v*) aqueous sodium hydroxide solution were added sequentially, mixing the contents after each reagent addition. The volume of the reaction mixture was then adjusted to 10 mL with deionized water.

A blank sample was prepared analogously, replacing the extract with deionized water. The results were expressed as quercetin equivalents (milligrams) per 100 g of mousse, calculated as the arithmetic mean of three analytical repetitions. The calibration curve equation used was y = 0.8506x + 0.0115, where y is the absorbance of the sample and x is the quercetin concentration in mg/L.

- The determination of antioxidant activity of the analyzed fruit mousses using a spectrophotometric method and DPPH assay

The antioxidant activity (AA) of the analyzed samples was determined using the method developed by Blois [96] based on the aqueous–methanolic extracts obtained from these products. For this purpose, 0.1 mL of the extract was mixed in a test tube with 3.0 mL of 1 mM DPPH radical solution previously diluted tenfold with methanol. After 60 min of incubation, the absorbance of the resulting mixture was measured at the wavelength of λ = 515 nm using a UV/Vis spectrophotometer (Jasco, Tokyo, Japan) against methanol. The results were expressed as millimoles of Trolox equivalents (TE) per 100 g of mousse, calculated as the arithmetic mean of three analytical replicates. The calculations were based on the calibration curve equation y = −0.881x + 1.0102, where y represents the absorbance of the tested sample and x represents the Trolox concentration in mM/L.

- The determination of the antioxidant activity of the analyzed fruit mousses using a spectrophotometric method and ABTS assay

The antioxidant activity (AA) of the analyzed samples was determined using the method developed by Baltrušaitytė et al. [97] based on the aqueous–methanolic extracts obtained from these products. For this purpose, 0.1 mL of the extract was mixed in a test tube with 6.0 mL ABTS cation radical solution previously diluted with phosphate buffer (pH = 7.4) in order to obtain the absorbance value of 0.80 ± 0.03. After 30 min of incubation, the absorbance of the resulting mixture was measured at the wavelength of λ = 734 nm using a UV/Vis spectrophotometer (Jasco, Tokyo, Japan) against phosphate buffer. The results were expressed as millimoles of Trolox equivalents (TE) per 100 g of mousse, calculated as the arithmetic mean of three analytical replicates. The calculations were based on the calibration curve equation y = −0.42308x + 0.788, where y represents the absorbance of the tested sample and x represents the Trolox concentration in mM/L.

- The determination of the reducing activity of the analyzed fruit mousses using a spectrophotometric method and FRAP assay

The reducing activity of the analyzed samples was determined using the method developed by Benzie and Strain [98] based on the aqueous–methanolic extracts obtained from these products. For this purpose, 3.3 mL of the acetate buffer (pH = 3.6) was mixed in a test tube with 0.33 mL of 20 mM ferric chloride (FeCl_3_) solution and 0.33 mL of 10 mM TPTZ (2,4,6-tris(2-pyridyl)-1,3,5-triazine) solution in 40 mM HCl solution and then incubated in a water bath at 37 °C. After incubation, 0.33 mL of the extract was added to the obtained mixture followed by incubation at 37 °C for 15 min. After cooling, the absorbance of the resulting mixture was measured at the wavelength of λ = 593 nm using a UV/Vis spectrophotometer (Jasco, Tokyo, Japan) against the blank sample including deionized water instead of the extract. The results were expressed as micromoles of ferrous ion (Fe^2+^) equivalents (TE) per 100 g of mousse, calculated as the arithmetic mean of three analytical replicates. The calculations were based on the calibration curve equation y = 0.001894x + 0.02159, where y represents the absorbance of the tested sample and x represents the ferrous sulphate (FeSO_4_) in µM/L.

- The procedure for the analysis of free phenolic compounds in the tested mousses using a chromatographic method.

Chromatographic analysis of the extracts was carried out using a high-performance liquid chromatography (HPLC) system (Jasco, Tokyo, Japan) equipped with a gradient pump, degasser, and diode array detector (DAD, Jasco, Tokyo, Japan). The column temperature was set at 30 °C, and the injection volume was 20 µL. The presence of hydroxybenzoic acids (i.e., gallic acid) and flavonoids (i.e., catechin and hesperidin) was identified at a wavelength of λ = 280 nm, whereas hydroxycinnamic acids (i.e., caffeic and p-coumaric acids) were detected at λ = 320 nm. Chromatographic separation was performed using an RP-18 column (Purospher, 250 mm × 4.6 mm, 5 µm particle size; Merck, Darmstadt, Germany), with a solvent flow rate of 1 mL/min. A gradient elution method was employed using two mobile phases, phase A (2.5% acetic acid solution in water, *v*/*v*) and phase B (gradient-grade acetonitrile), based on a previously described procedure [99]. The chromatographic analysis was performed as follows: During the first 10 min, a linear gradient was applied, followed by an increase in the proportion of phase B to 15, 20, 30, and 40% at 20, 30, 40, and 50 min, respectively. After completing the analysis, the chromatographic column was flushed isocratically with acetonitrile for 10 min prior to the next run.

Qualitative analysis was carried out by comparing the retention times of the obtained chromatographic peaks with those of the polyphenol standards acquired from Sigma (Steinheim, Germany). We also compared the absorption spectra of the detected peaks with the corresponding spectra of the standards.

Quantitative analysis was conducted using calibration curves prepared for the analyzed polyphenols, based on solutions within the concentration range of 0.02–0.2 mg/mL. Chromatographic measurements were performed in triplicate for both the analyzed samples and the polyphenol standards. For calibration curves, concentration values were calculated as the mean of three repetitions, whereas the polyphenol contents in the tested samples were expressed as the arithmetic mean of three replicate analyses.

- The procedure for the extraction and analysis of phenolic compounds present in bound form in the tested mousses using a chromatographic method (HPLC)

To analyze the presence and content of polyphenols occurring in bound form in the tested mousses (i.e., as esters and glycosides), the water–methanol extracts obtained from the samples were subjected to alkaline hydrolysis according to the procedure proposed by Nardini and Ghiselli [100]. For this purpose, 10 mL of the water–methanol extract was mixed with 90 mL of a hydrolyzing mixture (containing 1% ascorbic acid and 10 mM EDTA dissolved in a 2 M NaOH solution). The resulting mixture was incubated in a water bath at 30 °C under an inert gas atmosphere (argon). After completion of hydrolysis, the solution was cooled to room temperature and subsequently acidified with diluted hydrochloric acid (1:2, *v*/*v*) until pH = 2 was reached.

The extraction of phenolic compounds released from bound forms was performed according to the procedure previously described by Socha et al. [101]. For this purpose, the previously acidified polyphenol solution was saturated with sodium chloride at a ratio of approximately 300 mg per 1 mL of solution until saturation was reached. The obtained solution was extracted three times with ethyl acetate in a separatory funnel using an ethyl acetate-to-solution volume ratio of 1:3 for each extraction step. After extraction, the collected ethyl acetate fractions were combined and evaporated using a vacuum rotary evaporator at 35 °C under an inert gas atmosphere until a dry residue was obtained. The resulting solid residue was dissolved in 5 mL of methanol, transferred to a polypropylene tube, and stored at 5 °C until chromatographic analysis. Before HPLC determination, the methanolic extracts were purified by using solid-phase extraction (SPE), analogously to the procedure applied for the previous analysis (without alkaline hydrolysis).

#### 3.6.12. Microbiological Analysis

- Assessment of the microbiological purity of the products

Microbiological analyses were performed at two intervals, i.e., at day 0, 7, and 14 and after 6 months of storage (storage of tightly closed jars at room temperature in the dark). At each time point, representative samples were assessed in triplicate under sterile conditions. The examined mousses were surface-plated by spreading a loopful on the following general and selective media: Trypticase Soya Agar (TSA, incubation at 36 ± 1 °C and observation after 24, 48, and 72 h and 7 days) for total bacterial count, Malt Extract Agar (MEA, incubation at 25 °C and observation after 3, 5, and 10 and 15 days) for yeasts and molds, Baird–Parker agar (BP, incubation at 36 ± 1 °C and observation after 24, 48, and 72 h) for coagulase-positive Staphylococci, and SS agar (incubation at 36 ± 1 °C and observation after 24, 48, and 72 h) for *Salmonella* and *Shigella*. Plates were observed at each storage interval and after incubation period the colonies were counted and the results expressed and CFU/g of products.

The detection limit for all culture-based assays was 1 CFU/g of product, corresponding to the minimum count detectable by surface plating. The samples were analyzed immediately after opening; when storage prior to analysis was required, jars were kept tightly closed at room temperature in the dark and processed within 8 h. The long-term stability of the products was assessed after 6 months of storage under the same conditions.

- Assessment of the antibacterial activity of the products

The antibacterial effect was evaluated against various bacteria commonly associated with respiratory tract infections as well as those typical of upper respiratory tract microbiota, including *Klebsiella pneumoniae*, *Moraxella catarrhalis*, *Neisseria flavescens*, *Staphylococcus aureus*, *Staphylococcus epidermidis*, *Staphylococcus haemolyticus*, *Staphylococcus saprophyticus*, *Staphylococcus* spp., *Streptococcus anginosus*, *Streptococcus dysgalactiae*, *Streptococcus mitis*, *Streptococcus pyogenes*, *Streptococcus salivarius*, group C *Streptococcus*, group F *Streptococcus*, β-haemolytic *Streptococcus*, *Streptococcus pneumoniae*, and *Stenotrophomonas maltophilia*. In total, 38 bacterial strains were examined.

Mueller–Hinton Agar (MHA) plates were prepared and inoculated by evenly spreading a standardized bacterial suspension across the entire surface using a sterile swab. A sterile inoculation loop was used to apply a loopful (c.a. 0.05–0.1 g) of each of the three mousse samples directly onto the surface of the inoculated agar plates. Plates were incubated at 36 ± 1 °C for 24 h under aerobic conditions. After incubation, the presence and (if relevant) size of bacterial growth inhibition zones were measured in millimeters. The tests were conducted in three replicates. The results were expressed as the mean growth inhibition zone diameters ± standard deviation.

#### 3.6.13. Statistical Analyses

The data were subjected to statistical analysis using one-way analysis of variance (ANOVA) followed by Fisher’s test (*p* < 0.05) to determine significant differences between the tested mousse variants. The least significant differences between the means as well as Pearson’s linear correlation coefficients were calculated using software Statistica 13.0 (TIBCO, PaloAlto, CA, USA).

Statistical significance of differences in antimicrobial activity between formulations was assessed using ANOVA with Tukey’s post hoc test (*p* < 0.05).

## 4. Conclusions

In this study, the effectiveness of the applied encapsulation technique for the development of a fully natural food product was confirmed. The use of starch as the capsule wall material enabled effective protection of bioactive compounds, including elderberry fruit extract and grape seed oil, the latter being a source of unsaturated fatty acids. Scanning Electron Microscopy (SEM) analysis confirmed the presence of spherical submicron capsules with an average diameter of approximately 400–900 nm. FTIR-ATR spectroscopy further confirmed the incorporation of grape seed oil into the fruit mousse formulations. More importantly, spectral differences between F1 and F2 indicate that starch-based encapsulation modifies the interaction landscape among the formulation components, leading to stabilized and protected lipid dispersion. The combined evidence of increased carbonyl band intensity, alterations in the glycosidic region, and reduced water accessibility provides multifaceted confirmation that the encapsulation strategy effectively protected the oil within the starch matrix. These findings support the conclusion that starch-based nanocarrier systems represent a promising approach for improving oxidative stability and extending the functional performance of lipid-rich ingredients in food applications, particularly in systems with a high content of unsaturated fatty acids, where oxidative degradation remains a significant technical challenge. Despite the high water content and elevated water activity, the fortified fruit mousses maintained a low pH within a safe range, thereby supporting product stability and shelf life. The low total color difference values (ΔE* < 1) indicate that enrichment of samples F1 and F2 did not result in perceptible color changes compared to the control sample, confirming that the encapsulated additives can be incorporated without adversely affecting visual acceptability. Rheological and textural analyses demonstrated that all mousses exhibited a stable, gel-like structure (G′ > G″), while the addition of bioactive substances into the mousses (F1 and F2) enhanced their viscoelastic properties without significantly compromising texture parameters. These findings indicate that the applied encapsulation strategy enables structural reinforcement of the product while preserving desirable functional and sensory attributes. The capsule-enriched mousse (F2) exhibited significantly higher antioxidant and reducing activities, as determined by DPPH, ABTS, and FRAP assays, compared with the control sample (F0), indicating effective protection and preservation of the phenolic compounds which exhibit pro-health activity through encapsulation. Moreover, the higher concentrations of key phenolic compounds determined chromatographically, including catechin, caffeic acid, *p*-coumaric acid, and hesperidin, reflected by a strong correlations with the antioxidant parameters, confirm that encapsulation enhances the health-promoting potential of mousse F2 without deterioration of its functional quality. The analyzed fruit mousses also exhibited very good microbiological stability throughout the storage period, meeting safety criteria for ready-to-eat products and confirming the effectiveness of the applied processing and storage conditions. The observed antibacterial activity was selective and more pronounced against selected Gram-positive bacteria; however, enrichment of the mousses with elderberry extract (F1, F2) only slightly increased growth inhibition. These results suggest that naturally occurring phenolic compounds present in the fruit matrix play a predominant role in the antimicrobial activity of the studied mousses.

## Figures and Tables

**Table 1:** Water content, water activity, and pH of mousses.

Fruit Mousses	Water Content (%)	Water Activity a_w_ (−)	pH
F0	84.97 ± 0.55 ^b^	0.84 ± 0.01 ^a^	3.68 ± 0.07 ^a^
F1	85.14 ± 0.01 ^a,b^	0.80 ± 0.01 ^b^	3.78 ± 0.04 ^a^
F2	84.83 ± 0.20 ^a^	0.81 ± 0.00 ^b^	3.82 ± 0.02 ^a^

The values are expressed as the mean ± standard deviation. The presence of the same superscript letter (a, b) in each column indicates that there is no statistically significant difference between the values (*p* < 0.05).

**Table 2:** Color parameters of fruit mousses.

Fruit Mousses	L* (D65)	a* (D65)	b* (D65)	C*	h*	∆E*
F0	55.02 ± 0.06 ^c^	10.74 ± 0.38 ^b^	6.49 ± 0.08 ^b^	12.55 ± 0.34 ^b^	0.54 ± 0.01 ^a^	-
F1	55.15 ± 0.02 ^b^	11.18 ± 0.02 ^a^	6.75 ± 0.01 ^a^	13.06 ± 0.01 ^a^	0.54 ± 0.00 ^a^	0.53 ± 0.01 ^b^
F2	55.63 ± 0.02 ^a^	11.43 ± 0.03 ^a^	6.77 ± 0.05 ^a^	13.29 ± 0.05 ^a^	0.53 ± 0.00 ^a^	0.97 ± 0.03 ^a^

The values are expressed as the mean ± standard deviation. The presence of the same superscript letter (a, b, c) in each column indicates that there is no statistically significant difference between the values (*p* < 0.05).

**Table 3:** Textural parameters of fruit mousses.

Fruit Mousses	Hardness (N)	Adhesiveness (N·s)	Springiness (-)	Cohesiveness (-)	Gumminess (-)	Chewiness (-)
F0	0.40 ± 0.02 ^b^	9.77 ± 0.38 ^a^	0.97 ± 0.01 ^a^	0.84 ± 0.02 ^a^	0.34 ± 0.02 ^a,b^	0.33 ± 0.02 ^a,b^
F1	0.41 ± 0.02 ^a,b^	9.86 ± 0.32 ^a^	0.96 ± 0.00 ^a^	0.81 ± 0.02 ^a^	0.33 ± 0.02 ^b^	0.32 ± 0.01 ^b^
F2	0.43 ± 0.01 ^a^	10.30 ± 0.65 ^a^	0.96 ± 0.01 ^a^	0.83 ± 0.02 ^a^	0.36 ± 0.01 ^a^	0.34 ± 0.01 ^a^

The values are expressed as the mean ± standard deviation. The presence of the same superscript letter (a, b) in each column indicates that there is no statistically significant difference between the values (*p* < 0.05).

**Table 4:** Total polyphenol and flavonoid content and antioxidant and reducing activity of tested mousse samples.

Fruit Mousses	Total Phenolic Content [mg GAE/100 g]	Total Flavonoid Content [mg QE/100 g]	DPPH Assay TE [mM/100 g]	ABTS Assay TE [mM/100 g]	FRAP Assay Fe (II) [mM/100 g]
F0	73.163 ^a^ ± 1.408	1.088 ^a^ ± 0.098	0.3670 ^a^ ± 0.0036	1.0580 ^a^ ± 0.0412	0.9886 ^a^ ± 0.0173
F1	76.141 ^a^ ± 2.116	1.090 ^a^ ± 0.106	0.3889 ^b^ ± 0.0119	1.0779 ^a^ ± 0.0106	1.0288 ^b^ ± 0.0128
F2	78.636 ^a^ ± 1.785	1.040 ^a^ ± 0.035	0.4130 ^c^ ± 0.0051	1.1877 ^b^ ± 0.0128	1.0558 ^b^ ± 0.0139

The values are expressed as the mean ± standard deviation. The presence of the same superscript letter (a, b, c) in each column indicates that there is no statistically significant difference between the values (*p* < 0.05). GAE—gallic acid equivalents, QE—quercetin equivalents, TE—Trolox equivalents, Fe (II)—ferrous ions equivalents, and mM—millimoles.

**Table 5:** Content of phenolic acids and flavonoids present in free and bound forms determined by chromatographic analysis.

Content of Phenolic Compounds in Tested Mousses Present in Free Form (Analyzed Before Alkaline Hydrolysis) mg/100 g					
Fruit Mousses	Gallic Acid	Caffeic Acid	*p*-Coumaric Acid	Catechin	Hesperidin
F0	0.30562 ^a^ ± 0.02944	—	—	4.44377 ^a^ ± 0.20094	—
F1	0.29072 ^a^ ± 0.03589	—	—	6.55570 ^b^ ± 0.07204	—
F2	0.29044 ^a^ ± 0.02343	—	—	7.18214 ^c^ ± 0.23337	—
Total phenolic compound content in the mousses (mg/100 g) (including the sum of free and bound phenolic compounds)					
F0	0.30562 ^a^ ± 0.02944	0.88374 ^a^ ± 0.01623	0.53715 ^a^ ± 0.00824	4.44377 ^a^ ± 0.20094	0.27994 ^a^ ± 0.00167
F1	0.29072 ^a^ ± 0.03589	1.02426 ^b^ ± 0.00535	0.56104 ^a,b^ ± 0.03134	6.55570 ^b^ ± 0.07204	0.39335 ^b^ ± 0.01334
F2	0.29044 ^a^ ± 0.02343	1.06849 ^c^ ± 0.00181	0.59204 ^b^ ± 0.00873	7.18214 ^c^ ± 0.23337	0.43666 ^c^ ± 0.01690

The values are expressed as the mean ± standard deviation. The presence of the same superscript letter (a, b, c) in each column indicates that there is no statistically significant difference between the values (*p* < 0.05).

**Table 6:** Fruit mousse recipe.

Ingredients (g)	F0	F1	F2
strawberry	265	265	265
bananas	130	130	130
apple	605	605	605
starch gel	20	20	20
dry elderberry extract	0	1.0	1.0
grape seed oil	0	1.5	1.5
distilled water	0	1.5	1.5

## Data Availability

The data presented in this study are available on request from the corresponding author. The data are not publicly available due to privacy restrictions and ongoing research utilizing the same dataset.

## Abbreviations

The following abbreviations are used in this manuscript:

FTIR	Fourier Transform Infrared Spectroscopy
SEM	Scanning Electron Microscopy
CIE Lab*	Commission Internationale de l’Éclairage Lightness and Color Coordinates
TPC	Total Polyphenol Content
TFC	Total Flavonoid Content
HPLC	High-Performance Liquid Chromatography
AA	Antioxidant Activity
GAE	Gallic Acid Equivalents
QE	Quercetin Equivalents
TE	Trolox Equivalents
FRAP	Ferric ion Reducing Antioxidant Parameter
ABTS	2,2′-azinobis(3-ethylbenzothiazoline-6-sulfonic acid) radical cation decolorization assay
DPPH	2,2-diphenyl-1-picrylhydrazyl radical scavenging assay
ISO	International Organization for Standardization
PN-EN	Polish Standard-European Norm
